# Microbiological and hydrogeological assessment of groundwater in southern Italy

**DOI:** 10.1007/s10661-016-5655-y

**Published:** 2016-10-25

**Authors:** Osvalda De Giglio, Giovanna Barbuti, Paolo Trerotoli, Silvia Brigida, Angelantonio Calabrese, Giuseppe Di Vittorio, Grazia Lovero, Giuseppina Caggiano, Vito Felice Uricchio, Maria Teresa Montagna

**Affiliations:** 1 Department of Biomedical Science and Human Oncology—Hygiene Section, University of Bari Aldo Moro, Piazza G. Cesare 11—Policlinico, Bari, Italy; 2 Water Research Institute, National Research Council, Bari, Italy; 3 Prevention Department, Azienda Sanitaria Locale Provincia di Bari, Bari, Italy

**Keywords:** Groundwater, Microbiological pollution, Drinking water, Apulia, Italy, Fecal contamination

## Abstract

This study represents the first investigation of microbiological groundwater pollution as a function of aquifer type and season for the Apulia region of southern Italy. Two hundred and seven wells were randomly selected from those monitored by the Regional Agency for Environmental Protection for emergency use. Both compulsory (*Escherichia coli*, Total Coliform, and Enterococci) and optional (*Pseudomonas aeruginosa*, *Salmonella* spp., Heterotrophic Plate Count at 37 and 22 °C) microbiological parameters were assessed regularly at these wells. Groundwater from only 18 of the 207 (8.7 %) wells was potable; these all draw from karst-fissured aquifers. The remaining 189 wells draw from karst-fissured (66.1 %) or porous (33.9 %) aquifers. Of these, 82 (43.4 %) tested negative for *Salmonella* spp. and *P. aeruginosa*, while 107 (56.6 %) tested positive for *P. aeruginosa* (75.7 %), *Salmonella* spp. (10.3 %), or for both *Salmonella* spp. and *P. aeruginosa* (14 %). A logistic regression model shows that the probability of potable groundwater depends on both season and aquifer type. Typically, water samples were more likely to be potable in autumn-winter than in spring-summer periods (odds ratio, OR = 2.1; 95 % confidence interval, 95 % CI = 1.6–2.7) and from karst-fissured rather than porous aquifers (OR = 5.8; 95 % CI = 4.4–7.8). Optional parameters only showed a seasonal pattern (OR = 2.6; 95 % CI = 1.7–3.9). Clearly, further investigation of groundwater microbiological aspects should be carried out to identify the risks of fecal contamination and to establish appropriate protection methods, which take into account the hydrogeological and climatic characteristics of this region.

## Introduction

In recent years, many water-borne diseases from contaminated groundwater have been reported by countries with various levels of economic development (Zhang et al. 2014; Beer et al. 2015). These diseases are caused by pathogenic microorganisms of enteric origin, distributed by anthropogenic and natural processes, such as grazing, manure spreading, and uncontrolled sewage disposal. Runoff processes mobilize these microorganisms, leading to potential contamination of groundwater through soil infiltration. It is a widely held perception that groundwater quality, especially of deeper aquifers, is preserved by purification of this type of contamination through soil filtration processes, or is protected by overlying impermeable strata (O’Reilly et al. 2007). This perception is incorrect (Kyle et al. 2008). In fact, microbiological groundwater contamination occurs more easily in aquifers, in which permeability arises from fractures and karst phenomena (Berger 2008). In these aquifers, water can move quickly, allowing transport of microorganisms, with insignificant interaction between these microorganisms and the host rock. In porous aquifers, such as gravel or coarse sand aquifers, permeability results from pore space between grains. In this case, although the microorganisms can be easily transported (Berger 2008), they also undergo interaction with the sand-gravel matrix, which can reduce pollution loads.

To understand the dynamics that control the pathogenic contamination of groundwater, diverse factors must be taken into account. Both survival and persistence of microorganisms in groundwater are influenced by climate, rainfall, and temperature; such influences are particular to the microorganism involved (O’Dwyer et al. 2014; Engström et al. 2015). Meanwhile, the transport and the fate of such microorganisms can be determined by the hydrogeological characteristics of the aquifer (Bitton and Harvey 1992; Celico et al. 2004; Berger 2008; O’Dwyer et al. 2014). Furthermore, phenomena that regulate aquifer recharge (e.g., heavy rainfall), outflow, and discharge processes, as well as seawater intrusions, all affect microbiological pollution and make this issue difficult to manage (Bitton and Harvey 1992). Finally, groundwater is an important source for domestic, agricultural and industrial uses, and its intensive exploitation also can increase underground pollution (Shahbazi and Esmaeili-Sari 2009; Wu et al. 2015). Currently, it is well recognized that failure to protect groundwater sources, along with inadequate water treatment, are the primary reasons for bacterial contamination of drinking water (Pitkänen et al. 2011).

The current Italian regulations controlling the quality of drinking water are outlined in Legislative Decree no. 31 dated February 2, 2001 (D. Lgs 31/01). According to this legislation, water is considered potable, if the following microbiological parameters of fecal origin (compulsory parameters) comply with stringent guidelines: *Escherichia coli* (EC), Total Coliforms (TC), and Enterococci (ENT) have to be absent in a 100 ml sample. The presence of other microorganisms (*Salmonella* spp., *Pseudomonas aeruginosa*, and enteric virus) and a Heterotrophic Plate Count (HPC) at 22 and 37 °C are given as optional parameters for the assessment of water quality, but are required when local health authorities suspect a sanitary risk. In Italy, few studies dealing with the microbiological pollution of groundwater have been carried out (Celico et al. 2004; Migliorati et al. 2008; Lugoli et al. 2011), despite increasing risk. Although public health authorities have strong regulations about the distances between human and animal waste disposal sites and drinking water wells, with the intent to protect human health, they neglect any hydrogeological assessment (Berger 2008). This is compounded by the fact that data available for the Apulia region are limited to restricted territories and were often measured during different monitoring periods. Thus, microbiological and hydrogeological data for this region are geographically and temporally fragmented (Montagna et al. 1997; Lugoli et al. 2011; Ielpo et al. 2012; Bagordo et al. 2015).

This study comprises part of more extensive research on the groundwater pollution in the Apulia region. Here, we focus on bacteriological aspects as a function of both aquifer type and season to assess the impact of these factors on groundwater contamination.

## Materials and methods

### Geographical and hydrogeological characteristics of the region

Apulia covers 19,358 km^2^ and has 4 million inhabitants, mostly engaged in tourism, agriculture, and livestock rearing. The region spans about 350 km in southeast Italy, between the Adriatic and Ionian Seas. It has extensive coastal development along its 800 km coastline. It is characterized by a typical Mediterranean climate, with mild dry winters and hot summers. Rainfall is the largest source of groundwater recharge, but it is highly irregular throughout the year. Because of its geographical, climatic, and geological conditions (a mostly karst-fissured landscape), this region lacks significant surface water, with the exception of the Ofanto and Fortore Rivers, occurring in the northeast of the region. Other rivers are short and ephemeral. Natural lakes are few and are distributed along the coast.

**Fig. 1:**
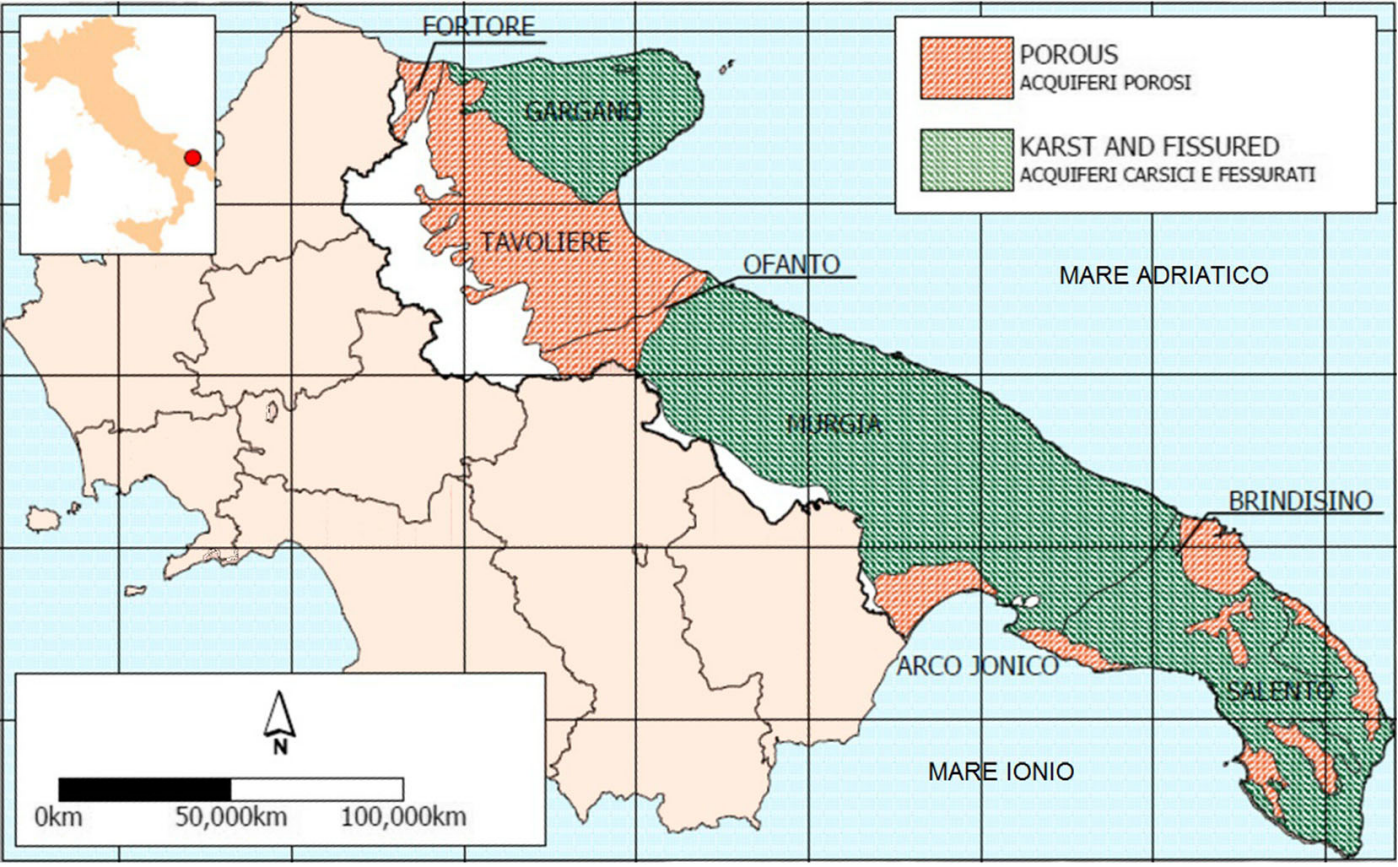
Distribution of aquifers in Apulia, southern Italy [from the Geological Survey of Italy]

In this region, the water supply is provided by Acquedotto Pugliese, a large Public Company that extends its activities over approximately 20,000 km^2^, drawing water from springs beyond regional boundaries (e.g., Basilicata and Campania), from artificial reservoirs (e.g., Occhito Lake), and from numerous groundwater wells. The groundwater wells are supplied by the Gargano, Murgia, and Salento karst-fissured aquifers (up to 400 m deep) and by the Tavoliere, Piana brindisina, and Arco Jonico Tarantino Occidentale (Arco Jonico) porous aquifers (less than 60 m deep) (Fig. 1).

Of the hydrogeological factors determining the spatial distribution of pollutants in the aquifers of Apulia, the coexistence of flow systems confined to small areas or spread throughout the region appears to be one of the most important. This characteristic causes outflow processes to have different lengths and depths in the same aquifer; thus, recharge occurs at different times (Maggiore and Pagliarulo 2004). A number of other relevant factors particular to groundwater in this region are outlined below.

In the Gargano and Murgia areas, groundwater aquifers are under pressure, except along a restricted coastal strip. Maximum piezometric levels, compared with sea level, are high in both areas. In Salento, subsurface water flows under phreatic conditions. This extensive underground aquifer is locally referred to as a ‘deep aquifer’ to distinguish it from other less important and less extensive ‘shallow aquifers’. The Tavoliere, Piana brindisina (a very small area compared with the rest of Apulia), and Arco Jonico areas are characterized by porous shallow aquifers, in which groundwater circulation is limited by significant clay formations.

### Sampling strategy

**Fig. 2:**
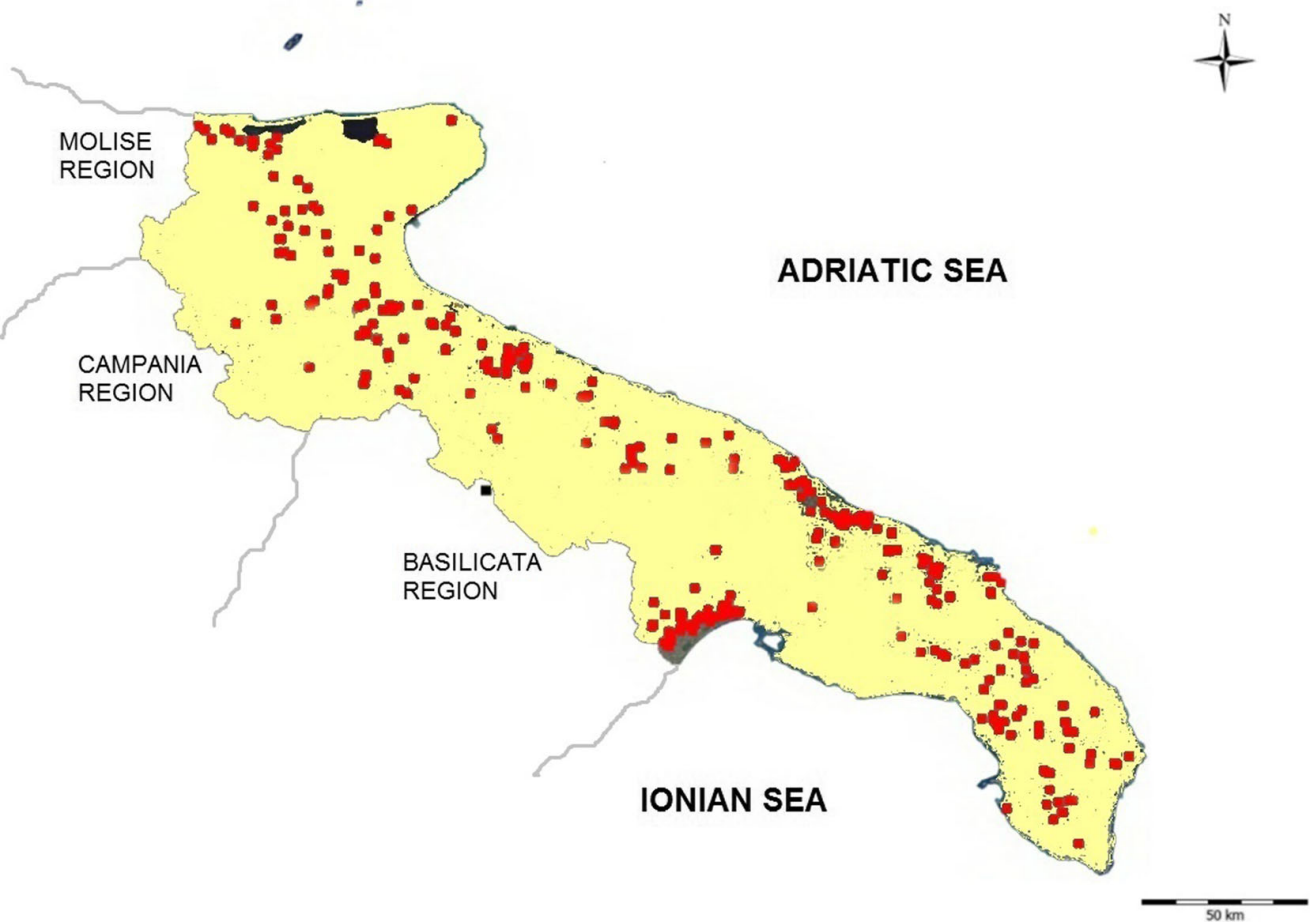
Distribution of monitored wells in Apulia, southern Italy

Sampling was carried out from January 2013 to December 2014. In total, 207 wells were randomly selected from those continuously monitored by the Regional Agency for Environmental Protection for use in emergencies (Fig. 2). Of these, 143 wells draw groundwater from karst-fissured aquifers and 64 from porous aquifers.

At each well, samples of 2 L of water were collected seasonally (four times a year), yielding 1656 samples. Water samples were collected in sterile containers between 9:00 am and 12:00 pm, under calm atmospheric conditions, with no rain (O’ Dwyer et al. 2014; Engström et al. 2015). The bottles were transported in a refrigerator (+4 °C) and processed within 5 h. To prevent any external contamination, the water was drawn through a tap that had been sterilized with a Bunsen burner. Groundwater was allowed to flow for 10 min before any sample was collected.

Tests for the following microorganisms were carried out: EC, TC, ENT, *Salmonella* spp., *P. aeruginosa*, together with a HPC at 22 and 37 °C.

#### *Escherichia coli* and Total coliform

A 100-ml aliquot of the water sample was filtered through a cellulose ester membrane (47 mm Ø and 0.45 μm-pore size; Millipore, Milan, Italy), placed on Tergitol 7-Triphenyltetrazolium chloride agar (Biolife Italiana srl, Milan, Italy) and incubated at 36 ± 2 °C for 24 ± 2 h. If no typical colonies were present, then the samples were incubated for an additional 24 ± 2 h. Lactose-positive colonies were subcultured onto a tryptone tryptophan medium (Sigma-Aldrich, St. Louis, MO, USA) and incubated at 37 ± 1 °C for 24 ± 2 h. If the resulting colonies were oxidase negative and indole positive, then they were assumed to be EC, while if they were oxidase negative, they were assumed to be TC (EN ISO 9308-1:^2000^).

#### Enterococci

A 100-ml aliquot of each water sample was filtered through a cellulose ester membrane (47 mm Ø and 0.45 μm pore size; Millipore, Milan, Italy). The membrane was placed over a Slanetz and Bartley agar medium (Biolife Italiana srl, Milan, Italy) and incubated at 36 ± 1 °C for 48 h. The colonies ranged in color from pink to dark red and brown, but only catalase and esculin hydrolysis positive ones were considered to be ENT (EN ISO 7899-2: 2003).

#### *Salmonella* spp.

A 1 L subsample of each water sample was filtered by a cellulose ester membrane (142 mm Ø and 0.45 μm pore size; Millipore, Milan, Italy). This membrane was placed in 100 ml sterile 0.1 % (*w*/*v*) peptone water (Thermo Scientific Oxoid, Milan, Italy) and homogenized for 1 min. Subsequently, an aliquot of the homogenized material was mixed with a selective enrichment medium, consisting of selenite cystine broth (Biolife Italiana srl, Milan, Italy). After incubation for 24 h at 35 °C, it was subcultured on two agar plates, brilliant green and xylose lysine deoxycholate (Becton Dickinson, Heidelberg, Germany), and incubated for another 24 h at 35 °C. From each plate, at least one colony suspected of being *Salmonella* was inoculated on triple sugar iron and lysine iron agar (Biolife Italiana srl, Milan, Italy), incubated for 24 h at 35 °C, and typed via specific serological tests (Standard Methods 9260D).

#### *Pseudomonas aeruginosa*

A 250-ml aliquot of the water sample was filtered through a cellulose ester membrane (47 mm Ø and 0.45 μm pore size, Millipore, Milan, Italy), placed over a *Pseudomonas* agar plate added to cetrimide and nalidix acid (Biolife Italiana srl, Milan, Italy), and incubated at 36 ± 1 °C for 48 h (EN ISO 12780:^2002^).

#### Heterotrophic plate count at 22 and 37 °C

Here, the agar inclusion technique was carried out. A 1-ml aliquot of the water sample was inoculated on two Petri dishes by adding 15 ml of Yeast Extract agar (Biolife Italiana srl, Milan, Italy) and incubated at 36 ± 1 °C for 48 h or at 22 ± 1 °C for 72 h (EN ISO 6222:^2001^).

### Definitions and limit values of bacteriological parameters

Water from a well was defined as potable, if all samples complied with the microbiological limits imposed by D. Lgs 31/01, i.e., EC, TC, and ENT, were absent in 100 ml. Water from a well was defined non-potable, if one or all parameters of fecal origin (EC, TC, and ENT) were present in at least one of the eight samples. We also used the optional parameters in our assessment: *Salmonella* spp. had to be absent in 1000 ml and *P. aeruginosa* absent in 250 ml. Although D. Lgs 31/01 sets no numeric value for HPC at 22 and 37 °C, it states that no “abnormal change” should be detected, when compared with values obtained during routine checks. We used these data as a guide to the general microbiological contamination of our samples.

## Data analysis

In this study, quantitative variables are given as median and interquartile ranges, because Gaussian distributions could not be assumed. Therefore, non-parametric Kruskal-Wallis tests were used for comparisons among independent groups. A Bonferroni correction was applied to *p* values to take into account multiplicity. Correlation among colony forming units (CFU) was carried out using the Spearman correlation coefficient (rs).

Qualitative variables are given as counts and percentages. The relationship between water drinkability as a dependent categorical dichotomous (Y/N) variable and independent factors was evaluated using a logistic regression model. Independent variables were typology of wells (karst-fissured vs. porous), sampling seasons (spring–summer vs. autumn–winter), and areas (each area vs. Arco Jonico). The selection of statistically significant variables was made with this logistic regression model, using the stepwise method and a *p* value of <0.05.

In this case, the outcome is the potability of the groundwater; hence, the odds ratio (OR) gives a measure of how the presence of a variable will affect the potability of the well water compared with the absence of that variable or at a chosen reference level. Thus, an OR > 1 means that a well has a greater chance of being suitable for drinking, if the variables have certain values with respect to chosen reference levels. All analyses were conducted using SAS 9.4 software.

## Results

### Bacteriological parameters

In terms of the compulsory parameters stated by D. Lgs 31/01, 18 out of 207 (8.7 %), wells yielded potable water*.* All these wells draw groundwater from karst-fissured aquifers, of which nine are from the Salento aquifer and nine are from the Murgia aquifer. EC, TC, and ENT were always absent, as well as *Salmonella* spp. and *P. aeruginosa*. The remaining 189 wells (91.3 %), drawing groundwater from karst-fissured (66.1 %) and porous (33.9 %) aquifers, revealed widespread contamination of fecal origin, i.e., EC, TC, and ENT always exceeded the D. Lgs 31/01 limits. Water from these 189 wells was considered non-potable*.* Water from 82 of these 189 wells (43.4 %) gave negative results for *Salmonella* spp. and *P. aeruginosa*, while water from 107 of the 189 wells (56.6 %) tested positive for *P. aeruginosa* (75.7 %), *Salmonella* spp. (10.3 %), and for both *Salmonella* spp. and *P. aeruginosa* (14 %).

The compulsory microbiological parameters stated by D. Lgs 31/01 showed a strong correlation. High levels of TC corresponded to high levels of ENT (rs = 0.75, *p* < 0.0001) and EC (rs = 0.79, *p* < 0.0001), while EC also was correlated with ENT levels (rs = 0.81, *p* < 0.0001). The presence of *Salmonella* spp. was significantly correlated with EC, TC, and ENT (rs = 0.15, *p* < 0.0001). *P. aeruginosa* was significantly correlated with EC and TC (rs = 0.13 and 0.15, respectively; *p* < 0.0001), but not with ENT (rs = 0.04, *p* = 0.0769).

The contamination levels, expressed in CFU, showed statistically significant differences with regard to aquifer type (Table 1). In particular, TC contamination in the karst-fissured aquifers of the Gargano area had a median of 30 CFU/100 ml and a range of 0–1500, while in porous aquifers in the Tavoliere area the groundwater contamination had a median of 20 CFU/100 ml and a range of 0–2000. This was significantly higher contamination than in other aquifers. Similarly, EC was highest in groundwater from wells drawing from the Gargano aquifer, with a median of 2 CFU/100 ml and a range of 0–800. ENT values were also highest for the Gargano aquifer, with a median of 3 CFU/100 ml and a range of 0–500.

**Table 1:** Median values of microbial indicators defined in D. Lgs 31/01

Microbial parameters	Karst-fissured aquifers (No. wells)						Porous aquifers (No. wells)				*p* value
	Gargano (21)		Salento (47)		Murgia (75)		Tavoliere (44)		Arco Jonico (20)		
	Median	Range	Median	Range	Median	Range	Median	Range	Median	Range	
Compulsory*											
Total coliform	30	0–1500	0	0–6000	0	0–2000	20	0–2000	16	0–6000	0.0014
*Escherichia coli*	2	0–800	0	0–3000	0	0–1800	0	0–900	0	0–1600	0.0014
Enterococci	3	0–500	0	0–900	0	0–800	1	0–500	0	0–600	0.0014
Optional**											
*Salmonella* spp	0	0–0	0	0–120	0	0–20	0	0–7	0	0–8	0.124
*P. aeruginosa*	0	0–800	0	0–3000	0	0–900	0	0–2000	0	0–600	0.06
HPC 22 °C	50	0–900	2	0–3000	3	0–22,000	50	0–6000	20	0–2300	0.0014
HPC 37 °C	25	0–700	0	0–3000	0	0–20,000	20	0–8000	9	0–2000	0.0014

*Total coliform, *Escherichia coli* and Enterococci (CFU/100 ml); **Salmonella spp. (CFU/L), *P. aeruginosa* (CFU/250 ml), and HPC (CFU/ml)

No statistically significant differences between the aquifers were found for *Salmonella* spp. and *P. aeruginosa* (*p* = 0.124 and 0.06).

In addition, HPC at 22 °C showed high contamination levels, especially for the Gargano aquifer (median = 50 CFU/ml, range = 0–900); the Tavoliere aquifer (median = 50 CFU/ml, range = 0–6000), and the Arco Jonico aquifer (median = 20 CFU/ml, range = 0–2300), with *p* < 0.0014. In contrast, HPC at 37 °C showed high contamination levels only in the Gargano aquifer (median = 25 CFU/ml, range = 0–700) and Tavoliere aquifer (median = 20 CFU/ml, range = 0–8000), with *p* < 0.0014.

### Factors influencing the groundwater quality

The logistic regression model showed that the probability that water from a well would be potable depends on season and aquifer type (Table 2). Considering the compulsory parameters, our water sample results show that groundwater was more likely to be suitable for drinking in autumn–winter than in spring–summer periods (OR = 2.1, 95 % CI = 1.6–2.7). The wells drawing from karst-fissured areas also were less contaminated than those drawing from porous areas (OR = 5.8, 95 % CI = 4.4–7.8). The water quality in terms of optional parameters was affected by season (OR = 2.6, 95 % CI = 1.7–3.9), but not by aquifer type (OR = 1.2, 95 % CI = 0.8–1.7).

**Table 2:** Results of logistic regression used to evaluate factors that affect water potability in the Apulia region

	UNIVARIATE			MULTIVARIATE		
	*p* value	OR	95 % CI	*p* value	OR	95 % CI
COMPULSORY PARAMETERS						
Season:						
autumn-winter vs. spring-summer	<0.0001	2.2	1.8–2.8	<0.0001	2.1	1.6–2.7
Aquifer:						
karst vs. porous	<0.0001	4.8	3.6–6.4	<0.0001	5.8	4.4–7.8
Area:						
Gargano vs. Arco Jonico	0.0003	1.5	0.8–3.1	Not entered		
Tavoliere vs. Arco Jonico	0.0011	1.9	1.1–3.6			
Murgia vs. Arco Jonico	<0.0001	11	6.3–19.4			
Salento vs. Arco Jonico	<0.0001	8.6	4.8–15.3			
OPTIONAL PARAMETERS						
Season:						
autumn-winter vs. spring-summer	0.0007	1.8	1.3–2.6	<0.0001	2.6	1.7–3.9
Aquifer:						
karst vs. porous	0.2703	1.2	0.8–1.7	Not entered		
Area:						
Gargano vs. Arco Jonico	0.339	0.8	0.4–1.6	Not entered		
Tavoliere vs. Arco Jonico	0.2112	0.8	0.5–1.5			
Murgia vs. Arco Jonico	0.2505	0.9	0.5–1.5			
Salento vs. Arco Jonico	0.0039	1.7	0.9–3.2			

The odds ratio (OR) expresses the probability of potability with respect to a reference class. The reference classes are spring–summer for season; porous for aquifer type; and Arco Jonico for area

## Discussion

In the Apulia region, groundwater is a clearly a fundamental resource for the local population because it is the principal source of drinking water and irrigation, given the absence of significant rivers or lakes. Cropland and livestock rearing occupy more than 70 % of its total area. Over time, the water demand has increased, especially in summer, linked to significant tourism. Seawater intrusion in coastal areas, caused by excessive groundwater withdrawal, together with local soil characteristics, especially filtration capacity, have affected groundwater quality, making it unusable (Lugoli et al. 2011; Ielpo et al. 2012).

It is a commonly held assumption that sandy or gravel aquifers hinder the spread of microorganisms, while karst-fissured aquifer are more vulnerable because flow through their fractured network reduces the contact time between bacteria and the surrounding media, decreasing the filtration capacity of the vadose zone, compared with sandy aquifers (Goeppert and Goldscheider 2011; Tryland et al. 2014). Groundwater contamination can be a particular concern in fractured and fissured aquifers, containing igneous, metamorphic, or carbonate rocks because groundwater flow can be relatively rapid in these aquifers. However, the same phenomenon can occur in highly permeable porous aquifers, such as moraine deposits, river gravels, and conglomerates (Berger 2008).

To the best of our knowledge, this study represents the first investigation of the microbiological pollution in groundwater throughout the Apulia region. Our data show that 91.3 % of the wells investigated, yielded water that was non-potable, and that porous aquifers were more contaminated than karst-fissured aquifers. The areas most contaminated were Tavoliere (porous aquifers) and Gargano (karst-fissured aquifers). Our data are not entirely in agreement with the relationships between pollution and hydrogeology described above, but we believe this may be the result of the local characteristics of these areas.

In particular, Tavoliere is characterized by a succession of permeable sandy–gravelly–stony soils, occasionally intercalated by less permeable silt and clay layers, which are facilitate microbiological contamination. Numerous torrential rivers cross this area; their flow is influenced by the intensity of rains and evapotranspiration. Consequently, this aquifer is shallow (25–50 m) and more exposed to local pollution linked to large-scale agriculture activities.

In contrast, Gargano has other factors influencing its groundwater quality, including similar seasonal and climatic factors, large tourist numbers, and intense agricultural land use (Idoko 2010; De Giglio et al. 2015).

The temporal analysis of our microbiological data highlights a seasonal pattern in both the D. Lgs 31/01 compulsory and optional indicator parameters, with higher than average pollution loads in spring and summer. Recent climate changes have led to a rise in temperature and to a more uneven rainfall throughout the year (Meteorological Service of the Italian Airforce). Consequently, cases of gastrointestinal diseases have become more frequent, because soil overflows are causing contamination of coastal and inland surface waters (Kim et al. 2013).

Our study of the D. Lgs 31/01 optional parameters revealed the presence of *Salmonella* spp., either alone or associated with *P. aeruginosa,* in 56.6 % of the wells unsuitable for drinking water. Other data on the occurrence of *Salmonella* spp. in groundwater within the study area are limited. D. Lgs 31/01 does not consider this bacteria to be a compulsory parameter, except when a sanitary risk is suspected, e.g., when contamination is linked to food-borne and/or water-borne diseases. Recent studies indicate that most *Salmonella* outbreaks are associated with community water systems and groundwater. Clearly, this represents a public health problem, even in industrialized countries. In such cases, water treatment was inadequate or insufficient (Franklin et al. 2009; Kozlica et al. 2010).

*P. aeruginosa* contamination shows a similar pattern to *Salmonella* contamination. Although this microorganism is found in natural waters, such as lakes and rivers, when there is heavy rain, they can infiltrate into subsurface waters and contaminate aquifers (Mena and Gerba 2009). *P. aeruginosa* is one of the most common etiological agents of infections, affecting the pulmonary tract, burns, and wounds (Mena and Gerba 2009); it can also colonize biofilms in domestic water systems (Asghari et al. 2013). We argue that it should be a compulsory monitoring parameter for water supplies to ensure the health of communities.

The colonies counted at 37 °C are used as an anthropogenic pollution index, with high numbers indicating an increased likelihood of fecal pollution. If undesirable changes in levels are reported, this should trigger additional inspections. In contrast, the colonies counted at 22 °C are an index of environmental pollution. Although this measure does not have any health implications, it allows us to highlight the presence of microbial species in the surface layers of soil that are easily adaptable to water (Ielpo et al. 2012).

## Conclusions

Our data highlight that local soil characteristics, especially its filtration capacity, can promote or hinder microbiological pollution. Climate characteristics, especially rainfall frequency, as well as human activities that involve any extensive use of water resources, influence the level of groundwater contamination, leading to reduced water availability and to progressive deterioration of its quality. We believe that management of groundwater quality requires a multidisciplinary approach focused on identifying the measures necessary to protect our water resources.
